# Predicting clinical outcomes in neuroblastoma with genomic data integration

**DOI:** 10.1186/s13062-018-0223-8

**Published:** 2018-09-27

**Authors:** Ilyes Baali, D Alp Emre Acar, Tunde W. Aderinwale, Saber HafezQorani, Hilal Kazan

**Affiliations:** 1 Department of Computer Engineering, Antalya Bilim University, Antalya, Turkey; 2 Electrical and Computer Engineering Graduate Program, Institute of Applied Sciences, Antalya Bilim University, Antalya, Turkey; 3 Graduate School of Informatics, Department of Health Informatics, Middle East Technical University, Ankara, Turkey; 4 Present Address: BC Cancer Agency Genome Sciences Centre, Vancouver, BC Canada; 5 Present Address: Department of Electrical and Computer Engineering, Boston University, Boston, US; 6 Present Address: Department of Computer Science, Purdue University, West Lafayette, US

**Keywords:** Neuroblastoma, Data integration, Cancer subtypes, Kernel k-means

## Abstract

**Background:**

Neuroblastoma is a heterogeneous disease with diverse clinical outcomes. Current risk group models require improvement as patients within the same risk group can still show variable prognosis. Recently collected genome-wide datasets provide opportunities to infer neuroblastoma subtypes in a more unified way. Within this context, data integration is critical as different molecular characteristics can contain complementary signals. To this end, we utilized the genomic datasets available for the *SEQC* cohort patients to develop supervised and unsupervised models that can predict disease prognosis.

**Results:**

Our supervised model trained on the *SEQC* cohort can accurately predict overall survival and event-free survival profiles of patients in two independent cohorts. We also performed extensive experiments to assess the prediction accuracy of high risk patients and patients without MYCN amplification. Our results from this part suggest that clinical endpoints can be predicted accurately across multiple cohorts. To explore the data in an unsupervised manner, we used an integrative clustering strategy named multi-view kernel k-means (MVKKM) that can effectively integrate multiple high-dimensional datasets with varying weights. We observed that integrating different gene expression datasets results in a better patient stratification compared to using these datasets individually. Also, our identified subgroups provide a better Cox regression model fit compared to the existing risk group definitions.

**Conclusion:**

Altogether, our results indicate that integration of multiple genomic characterizations enables the discovery of subtypes that improve over existing definitions of risk groups. Effective prediction of survival times will have a direct impact on choosing the right therapies for patients.

**Reviewers:**

This article was reviewed by Susmita Datta, Wenzhong Xiao and Ziv Shkedy.

**Electronic supplementary material:**

The online version of this article (10.1186/s13062-018-0223-8) contains supplementary material, which is available to authorized users.

## Background

Neuroblastoma is the second most common solid tumor in childhood. The disease can have a large variety of clinical outcomes ranging from spontaneous regression to relentless progression despite extensive therapies. As such, accurate prediction of disease prognosis is critical to improve the choice of therapies. MYCN gene is a well-established prognostic marker in neuroblastoma. Chromosomal amplification of the MYCN locus occurs in 25% of all neuroblastomas and is associated with poor prognosis [1]. However, patients without MYCN amplification may also have a poor outcome. Apart from MYCN amplification, a limited set of additional variables such as age at diagnosis, stage of disease etc. are used to stratify patients into distinct risk groups. Current risk group definitions are problematic as patients within the same risk group can still show variable prognosis. For instance, some low- or intermediate-risk patients still die from the disease whereas some high-risk patients show spontaneous regression. One promising direction is to utilize the recently available genomic datasets to discover complex molecular markers that can improve patient stratification. Several studies have been recently published along this line. For instance, Oberthuer et al. proposed a classifier that consists of 144 genes and showed its prognostic value using two cohorts of size 77 and 440 [2, 3]. Asgharzadeh et al. aimed to improve the stratification of metastatic neuroblastomas that lack MYCN gene amplification using a classifier based on 55 genes [4]. Similarly, Vermeulen et al. inferred a gene set signature from 30 training samples, and evaluated this signature with a test set of 313 samples and a validation set of 236 additional tumours [5].

Recently, several methods have been developed to integrate multiple genomic data for cancer subtype discovery [6–9]. In neuroblastoma literature, however, a large majority of the previous research have focused on one type of gene expression data (e.g., microarray) to infer molecular markers. Here, we utilized the diverse data types provided by the Sequencing Quality Control Consortium (*SEQC*) cohort (i.e., neuroblastoma challenge in Critical Assessment of Massive Data Analysis (CAMDA) 2017) to develop statistical models that can predict clinical outcomes in neuroblastoma. Using a linear Support Vector Machine (SVM, [10]), we were able to achieve a performance that is very close to the best reported performance in Zhang et al. for the supervised learning problem [11]. We also trained this model on the whole *SEQC* cohort and predicted overall survival (OS) and event-free survival (EFS) variables in two independent cohorts. Our results indicate that predicting OS and EFS is more challenging for high risk (HR) patients. We observed that focusing on only high risk patients during training improves the prediction accuracy of HR patients. In the last part of the study, we employed an unsupervised learning strategy based on kernel k-means. We used MVKKM to integrate multiple data types with the aim to identify subgroups that have significantly diverse survival profiles. We observed that integrating all gene expression datasets (i.e., RNA-seq and microarray data) improves over using these datasets individually. Also, integrating these data types with learned weights is a better option than integrating them uniformly. We also confirmed that the Cox regression model that uses our identified clusters as covariates yields a better model compared to the regression model with existing high risk / low risk (LR) labels. We repeated these analyses for the subset of patients that have Array Comparative Genomic Hybridization (aCGH) data, and reached similar conclusions. Altogether, our results suggest that utilizing genomic characterizations of tumors improve over current definitions of risk groups.

## Methods

### Data

RNA-seq, microarray and aCGH datasets for the *SEQC* cohort were downloaded from CAMDA website. Chen Suo and her colleagues have identified a potential mislabeling problem between normal and tumor samples when they compared the aCGH data against the MYCN status derived from FISH experiments (personal communication). Based on this comparison, the sign of the intensity values for 32 patients were reversed. The list of these patient ids can be found in Additional file 1. We used two versions of the RNA-seq data: *SEQC_NB_MAV_G_log2.txt* downloaded from CAMDA website and GSE62564_SEQC_NB_RNASeq_log2RPM.txt downloaded from GEO website for entry GSE62564. The data for the Versteeg cohort (GSE16476 [12]) were downloaded from R2 database (http://r2.amc.nl), and the data for the TARGET cohort were downloaded from the following link: https://ocg.cancer.gov/programs/target/data-matrix. Note that gene-level expression measurements were used for all our experiments. We used the *limma* package in R to perform differential expression analysis with RNA-seq MAV data [13]. *limma* outputs adjusted *p*-values that are corrected for multiple testing using the Benjamini-Hochberg method [14]. *seaborn.clustermap* function, (https://seaborn.pydata.org/generated/seaborn.clustermap.html) in Python (single linkage, Euclidean distance, standard scale = 1) was used to generate the heatmap.

### Supervised learning

We used the Support Vector Classification (SVC, http://scikit-learn.org/stable/modules/generated/sklearn.svm.SVC.html) function available in Python’s scikit-learn library for training SVM models. Feature selection is performed with the *sklearn.feature_selection.SelectKBest* method, (http://scikit-learn.org/stable/modules/generated/sklearn.feature_selection.SelectKBest.html) that uses ANOVA F-values to rank the features. The only parameter that needs to be tuned is the C parameter that determines the cost of misclassification. Models with lower values of C allow for more errors and learn a large margin whereas models with higher values of C aim to classify all examples correctly and learn a small margin. We considered the values {10^6^,10^5^,10^4^,10^3^,0.1,0.5,1,2,5,10,20,50,100,1000} for the C parameter and chose the one with the best accuracy within a nested cross-validation framework. As clinical endpoints (i.e., OS, EFS) have an unbalanced distribution in neuroblastoma cohorts, we further checked whether using class-specific weights improves the performance. Namely, we set the *class-weight* parameter to *balanced* so that the C parameter of each class is multiplied with a weight value that is inversely proportional to the corresponding class frequency in the input data. Nested cross-validation was used to decide between setting this parameter to *balanced* or to *none*.

### Unsupervised learning

Multi-view clustering methods aim to integrate the complementary information present in different views as this could enable the investigation of a complex system from different angles and levels. Within the cancer subtyping problem, the hypothesis is that combining different molecular characteristics of the same disease should give more comprehensive insights about the disease than considering a single characteristic type. MVKKM is an example of these approaches [15] that is closely related to unsupervised multiple kernel k-means [16]. Multiple kernel learning is concerned with using multiple kernels for the same data type whereas multi-view learning focuses on integrating different data types or views. In this approach, each view is transformed using a kernel function. Multiple kernels obtained from different views are then combined together with weights to derive the composite kernel ($\tilde {K}$):

1 $$ \tilde{K} = \sum\limits_{v=1}^{V} w_{v}^{p} K^{(v)}   $$

where *v* is the view index, *K*^(*v*)^ is the kernel matrix for view *v*, *w*_*v*_ indicates the weight of the kernel for view *v* and *p* is an exponent to control sparsity and needs to be fixed a priori. Assuming that p >= 1, the greater the p value, the less sparse the view weights become. This formulation can be easily extended to the case where there are multiple kernels calculated from the same view. The method iterates through two steps. In the first step, the individual kernels have to be combined to derive the composite kernel as explained above. In the second step, kernel k-means is applied on the composite kernel to infer the clustering assignment.

We used the code provided by Tzortzis et al. [15] with the Radial Basis Function (RBF) kernel. RBF kernel between two samples x and y can be calculated as *K*(*x*,*y*)=*exp*(−*γ*||*x*−*y*||^2^). To avoid selecting a specific *γ* parameter, we used six different *γ* parameters (i.e. {2^−14^,2^−15^,2^−16^,2^−17^,2^−18^,2^−19^}) for each view. Among the set of values {1.5,2,2.5}, 1.5 was chosen as optimal for the sparsity parameter p based on mean silhouette score [17]. Silhouette score for sample *i* is calculated as (*b*_*i*_−*a*_*i*_)/*max*(*a*_*i*_,*b*_*i*_) where *a*_*i*_ is the average distance of sample *i* to all other samples in sample *i*’s cluster and *b*_*i*_ is the average distance of sample *i* to all the samples in the closest cluster that *i* is not a part of. Lastly, silhouette scores of all data points are averaged to determine the mean silhouette coefficient. The number of clusters was determined by aggregating three measures: mean silhouette score, DUNN index [18] and connectivity [19]. A weighted rank aggregation method named RankAggreg [20] was used to combine the rankings obtained by these three metrics. We ran MVKKM multiple times with random initialization due to the local optima problem in k-means. The most frequent k value among the multiple runs was identified as the number of clusters.

### Predictive performance of supervised and unsupervised models

We used the *survival* package in R to perform Kaplan-Meier analysis. Because more than 80% of the *SEQC* data is right-censored, we applied the Cox proportional hazards regression analysis with Firth’s correction (hereafter named Coxphf) using the R package [21]. To compare between different Cox models, Bayesian Information Criterion (BIC) is calculated with the formula −2*loglik*+*mln*(*n*) where *loglik* is the log likelihood of the regression model, *n* is the number of samples and *m* is the number of parameters [22]. As an additional evaluation strategy, accelerated failure time models (AFT) was used. An iterative imputation procedure [23] was applied to handle right censoring. Root mean squared error adjusted for censoring (rmse, [24]) and Harrell’s c-index [25] were used as performance metrics. Harrell’s c-index calculates the frequency of concordant pairs where a pair of patients is called concordant if the patient with the higher risk prediction experiences the event before the other patient. Harrell’s c-index ranges between 0 and 1 and higher values correspond to more accurate prediction models. More details of the AFT model and the evaluation procedure can be found in [26]. The reported values are average of running 10-fold cross validation ten times.

## Results and discussion

### Validation of the *SEQC* model on independent cohorts

Table 1 shows statistics about the patients and the data types of the three cohorts that we worked with. We first performed supervised learning using SVM within the *SEQC* dataset. The mean cross-validation accuracy of the models that predict OS (i.e., occurrence of death from disease) and EFS (i.e., occurrence of progression, relapse or death) labels is close to the best accuracy reported for the same dataset [11] (for OS: our accuracy: 0.83 vs best accuracy: 0.85 and for EFS: our accuracy: 0.78 vs best accuracy: 0.78). In addition to the linear kernel, we also tried the RBF kernel for the SVM model; however, this resulted in no improvement in prediction accuracy. Besides, we tried gradient boosting and random forest models with no increase in performance. Altogether, these results suggest that the signal present in the *SEQC* cohort can be sufficiently captured by linear models.

**Table 1:** Patients and the data types for the cohorts: *SEQC*, *Versteeg*, *TARGET*

	SEQC	Versteeg	TARGET
# of patients	498	88	247
HR=1/HR=0	175/323	36/52	217/30
OS=1/OS=0	105/393	30/58	140/107
EFS=1/EFS=0	183/315	34/54	156/91
MYCN amp. / not amp.	92/401	16/72	68/175

Note that our HR definition is based on Children’s Oncology Group. The number of total patients do not add up to 498 for MYCN amplification label as there is missing data

#### Predicting outcome in Versteeg cohort

We used our model trained on the *SEQC* dataset to predict OS and EFS profiles of patients in an independent cohort that is called *Versteeg* dataset hereafter. This dataset includes the gene expression measurements and clinical data for 88 patients. Table 2 summarizes our results on the *Versteeg* data where we used Area Under the receiver operating characteristic curve (AUROC) and balanced accuracy as performance metrics. Several interesting observations can be derived from these results. First, when we compare the results of predicting OS and EFS labels, we observe that we predict OS more accurately than EFS in *All*→*All* and *HR*→*HR* contexts. Surprisingly, EFS prediction is more accurate when we focus only on patients with no MYCN amplification. Here we should note that some of the models listed in Table 2 use gene expression data derived from different platforms (e.g. RNA-seq or microarray). As such, we checked whether these observations still hold if we restrict the comparison to models that use gene expression data from the same platform (Additional file 4). The optimal models identified for OS and EFS prediction in *All*→*All* and *HR*→*HR* contexts use gene expression data from the same platform whereas this is not the case for *MYCN*_*NA*→*MYCN*_*NA* context. For *MYCN*_*NA*→*MYCN*_*NA* context, if we compare models that are derived from the same platform only, we still observe that EFS prediction has better performance than OS prediction.

**Table 2:** Predicting OS and EFS in *Versteeg* cohort using models trained from *SEQC* cohort

	OS			EFS		
Training → Test	Type	AUROC	Balanced Accuracy	Type	AUROC	Balanced Accuracy
All → All	Microarray C=0.001 Balanced	0.961	0.899	Microarray C=0.001	0.922	0.858
All → All (only HR patients)		0.847	0.717		0.897	0.751
HR → HR	RNA-seq (RPM) C=1000 Balanced	0.783	0.793	RNA-seq (MAV) C=1000 Balanced	0.736	0.613
MYCN_NA →MYCN_NA	RNA-seq (MAV) C=1000 Balanced	0.869	0.710	Microarray C=0.0001 Balanced	0.885	0.815

The first column displays the details about the training and test sets. *All*→*All* indicates that we used the whole *SEQC* data for training and the whole *Versteeg* data for testing. *All*→*All* with only HR patients corresponds to the same model as *All*→*All*; however, here the performance metrics are only calculated for HR patients. In the third row, we used only the HR patients within the SEQC data for training and similarly we tested only on HR patients within the *Versteeg* data. In the last row, we only consider the patients with no MYCN amplification for training and testing sets. The *Type* column indicates the details of the chosen model. For gene expression, microarray data and two versions of the RNA-seq data were used. As such, this entry shows the type of the gene expression data used for the best trained model. The same entry also includes the C parameter of the SVM model and the type of the class weights (balanced or uniform)

We investigated whether using only HR patients for training improves the prediction performance of OS and EFS of HR patients. Indeed, this is the case for OS prediction as balanced accuracy increases from 0.71 to 0.79. However, the same effect is not seen in predicting EFS. To confirm that the differences in accuracy between *All*→*All*and *HR*→*HR* contexts are indeed due to the different training datasets used, we repeated the comparison with models that use gene expression data from the same platform. When RNA-seq based gene expression data is used, models that are learned from only HR patients perform better than models that are learned from all patients in predicting OS of HR patients. However, we do not observe the same improvement for microarray based models. We also checked whether accounting for unbalanced class labels improves the performance. Turning on the *balanced* option for class weights gives a better model in terms of training set performance except for EFS prediction in *All*→*All* model. This could be due to the fact that the EFS label is less unbalanced than the OS label in the *SEQC* cohort.

**Fig. 1:**
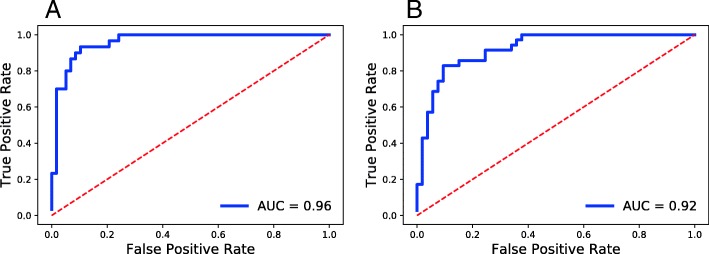
ROC curves for predicting clinical outcomes in *Versteeg* cohort **a** OS **b** EFS

**Fig. 2:**
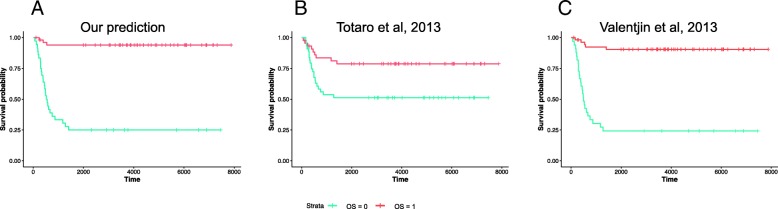
Comparison of the survival profiles of different methods that predict OS in *Versteeg cohort***a** Our prediction, log-rank test *p*-value: 3.6e-12 **b** Prediction made in Totaro et al., 2013 [27], log-rank test *p*-value: 0.007, **c** Prediction made in Valentjin et al., 2012 [28], log-rank test *p*-value: 7e-12

Figure 1 shows the ROC curves for predicting OS and EFS profiles. These curves reveal that we can predict OS with a high accuracy (i.e., AUROC: 0.96 and balanced accuracy: 0.89). We compared this performance with two different studies that aimed to predict OS on the same data. The first study is by Totaro et al. that focused on the IL6 gene and used its expression to classify patients into two groups [27] which results in a balanced accuracy of 0.65. The second study is from Versteeg group that aimed to predict neuroblastoma outcome irrespective of MYCN amplification [28]. To this end, they identified 157 genes as downstream targets of MYCN. They also used the *Versteeg* cohort data itself to confirm that the expression profile of these genes correlate with MYCN mRNA levels. Using these 157 genes, they were able to predict OS in *Versteeg* cohort with a balanced accuracy of 0.84. Figure 2 This result indicates that our model which is trained entirely on another cohort (i.e., *SEQC*) performs remarkably well on predicting OS in *Versteeg* cohort.

#### Predicting outcome in TARGET cohort

**Fig. 3:**
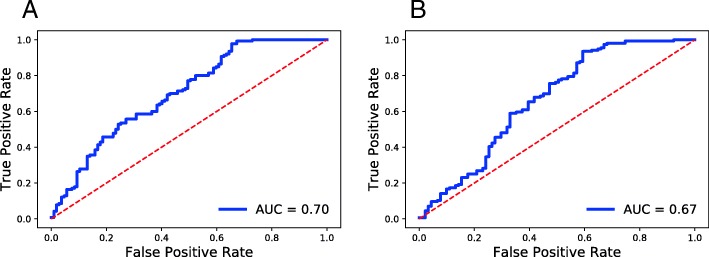
ROC curves for predicting clinical outcomes in *TARGET* cohort **a** OS **b** EFS

We repeated the experiments that we performed with *Versteeg* cohort for the *TARGET* cohort. (Figure 3 and Table 3). One important aspect of the *TARGET* cohort is the high fraction of HR patients. We should note that the models that we trained from *SEQC* cohort are different between Tables 2 and 3 as the genes common between *SEQC* and *Versteeg* cohorts are different than the genes that are common between *SEQC* and *TARGET* cohorts. As a first observation, we see that the accuracy of predicting OS and EFS is lower compared to our results on the *Versteeg* cohort. This is likely due to the different composition of HR and LR patients between the *SEQC* and *TARGET* cohorts. The accuracy of predicting both OS and EFS of HR patients increases when training is performed only with HR patients (comparing rows 2 and 3 in the table). This result is likely due to the high fraction of HR patients in *TARGET* cohort. When we restrict the comparison to models that use the same type of gene expression data, models derived from RNA-seq data predict both OS and EFS of HR patients more accurately when the training was performed with HR patients only (Additional file 4). Unlike our results on *Versteeg* cohort, predicting EFS is more accurate than predicting OS in terms of balanced accuracy. The optimal models identified for OS and EFS prediction in $All{\rightarrow } All$ context use gene expression data from the same platform whereas this is not the case for *HR*→*HR* and *MYCN*_*NA*→*MYCN*_*NA* contexts. For these two contexts, balanced accuracy of predicting EFS is higher than that of predicting OS even when we compare models that are derived from the same platform (Additional file 4).

**Table 3:** Predicting OS and EFS in *TARGET* cohort using models trained from *SEQC* cohort

	OS			EFS		
Training →Test	Type	AUROC	Balanced Accuracy	Type	AUROC	Balanced Accuracy
All →All	Microarray C=0.001 Balanced	0.703	0.592	Microarray C=0.0001 Balanced	0.666	0.594
All →All (only HR patients)		0.595	0.566		0.522	0.524
HR →HR	RNA-seq (RPM) C=1000 Balanced	0.611	0.579	Microarray C=0.0001 Balanced	0.570	0.612
MYCN_NA $\rightarrow $ MYCN_NA	RNA-seq (RPM) C=1000 Balanced	0.803	0.575	Microarray C=0.0001 Balanced	0.715	0.632

Column descriptions are same as Table 2

### Comparison of the selected features across different models

We investigated the 1000 selected features across the different models that we trained. First, we compared the OS and the EFS models that we used to predict *Versteeg* and *TARGET* cohorts. The number of genes that are common between OS and EFS models is 796 for *Versteeg* cohort and 793 for *TARGET* cohort. On the other hand, the overlap between the OS models trained with all patients and those trained with HR patients is only 88 for the *Versteeg* cohort and 86 for *TARGET* cohort. We observe similar numbers for the EFS models: 51 for *Versteeg* cohort and 31 for *TARGET* cohort. The overlap between the OS models trained with all patients and those trained with patients with no MYCN amplification is much higher: 384 for *Versteeg* cohort and 418 for *TARGET* cohort. The overlaps are even higher for EFS prediction: 783 for *Versteeg* cohort and 788 for *TARGET* cohort. These results are expected as the patients with no MYCN amplification form a larger subset of all patients when compared to HR patients. Finally, we also compared models trained with different cohorts. The OS model trained from *SEQC* cohort and the model trained from *Versteeg* cohort share 374 genes suggesting that the gene signature associated with OS is highly overlapping across cohorts. We observed a similar overlap (i.e., 278) for the *TARGET* cohort. The OS models trained only with HR patients showed a lower overlap size: 70 between *SEQC* and *Versteeg* cohorts and 81 between *SEQC* and *TARGET* cohorts. The smaller size of the HR training datasets is likely to be associated with these small overlaps.

### Predicting outcome in SEQC cohort

The experiments were performed in the opposite direction where we trained models using the *Versteeg* or *TARGET* cohorts and tested on the *SEQC* cohort. The linear SVM model trained on the *Versteeg* cohort predicts OS in *SEQC* cohort with a balanced accuracy of 0.80 and an AUROC of 0.86. This analysis reveals that even a small number of patients (i.e., *Versteeg* cohort: 88 patients) is satisfactory to learn a gene signature that can predict OS in a much larger cohort (i.e., *SEQC* cohort: 498 patients). Predicting EFS resulted in a similar accuracy (balanced accuracy: 0.75 and AUROC: 0.81). We repeated the same experiments where *TARGET* cohort is used as the training set. As expected, the predictive accuracy was lower compared to training on the *Versteeg cohort* (OS: bal. accuracy is 0.77 and AUROC is 0.86 and EFS: bal. accuracy is 0.69 and AUROC is 0.7). Though, we see the opposite pattern when we try to predict the outcome of HR patients using only HR patients as the training data. Namely, *TARGET*-trained models (OS: bal. accuracy is 0.59 and AUROC is 0.73) achieve a better predictive accuracy than *Versteeg*-trained models (OS: bal. accuracy is 0.58 and AUROC is 0.61). This is likely related to the much larger set of HR-only training data in *TARGET* cohort (*TARGET*: 217 vs *Versteeg*: 36).

### Unsupervised learning approaches for patient stratification

In addition to supervised learning approaches to predict OS and EFS in *SEQC* and other cohorts, we also investigated integrative clustering approaches to identify neuroblastoma subtypes in an unsupervised manner. To this end, MVKKM method was utilized which can cluster samples by integrating multiple data types. In MVKKM method, each data type is considered as a view, and multiple kernels can be used to represent each view. Because the relevance of the different views (or even the relevance of the different kernels in the same view) to the clustering task can vary, MVKKM learns a weight distribution across the kernels. This weight distribution enables the contribution of different kernels in varying degrees. Also, clustering the samples in the kernel space provides flexibility in applying non-linear feature transformations.

First, we applied MVKKM to all the patients to integrate different types of gene expression data. We used six RBF kernels with different gamma parameters for each view (See Methods). Table 4 compares the clusters obtained by using different datasets: (i) RNA-seq (MAV);(ii) RNA-seq (RPM); (iii) RNA-seq data (MAV and RPM); (iv) microarray data only ;(v) RNA-seq data and the microarray data (three views). We also included results for integration of RNA-seq and microarray datasets with uniform weights rather than MVKKM-learned weights. In all cases, the number of clusters was chosen as 2 based on mean silhouette score. To compare different patient stratification models, we used Coxphf and AFT models. We included the identified clusters, age and stage information as covariates in Coxphf and AFT regression models. For Coxphf, the log-likelihood of the model is used to calculate the BIC values. For AFT models, rmse and Harrell’s c-index are used for model evaluation. We observe that integrating the three types of gene expression data is better than using these datasets individually. These results also reveal that the integration of these datasets with MVKKM gives a better model than combining the kernels uniformly. When we sum the MVKKM-learned weights across the six kernels for RNA-seq (MAV), RNA-seq-(RPM) and microarray views, we obtain 0.31, 0.34 and 0.35 respectively. These weights indicate that RNA-seq-RPM and microarray datasets are more relevant for clustering neuroblastoma patients.

**Table 4:** Comparison of the clusters obtained with different data types from all patients

Model		Coxphf			AFT	
Data	k	HR (confidence interval)	Wald-test *p*-value	BIC	rmse	c-index
RNA-seq (MAV)	2	0.21 (0.11-0.40)	4.2e-07	1206	20.6	0.882
RNA-seq (RPM)	2	3.34 (1.82-6.50)	5.2e-05	1215	21.2	0.882
RNA-seq datasets	2	3.46 (1.89-6.70)	2.5e-05	1214	22.4	0.883
Microarray	2	0.26 (0.15-0.42)	5.8e-09	1197	32.0	0.870
All datasets	2	0.16 (0.07-0.30)	8.3e-10	1193	19.0	0.887
All datasets (uniform)	2	0.15 (0.07-0.31)	4.4e-09	1197	19.9	0.886
High Risk / Low Risk	2	0.45 (0.20-0.96)	8.5e-07	1228	10.3	0.885

**Fig. 4:**
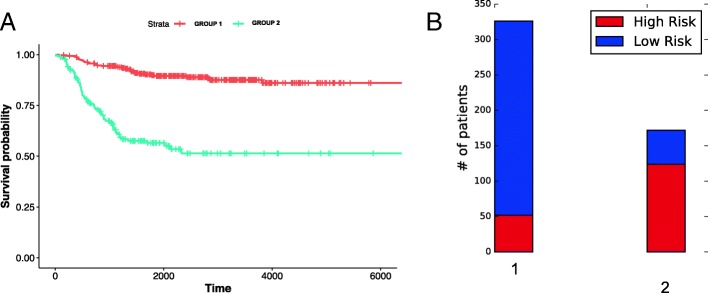
Analysis of the clusters obtained with both RNA-seq and microarray datasets **a** Kaplan-Meier analysis (log-rank test *p*-value: 3.9e-20) **b** Distribution of low risk and high risk patients in identified clusters

Figure 4a shows the Kaplan-Meier analysis of obtained clusters when both RNA-seq and microarray datasets are used. The log-rank test p-value equals to 3.9e-20 confirms that the patients in the two clusters show distinct prognosis. We also investigated the fraction of LR and HR patients in our obtained clusters (Fig. 4b). A larger proportion of cluster 1 patients are LR patients; however, HR patients still exist. The opposite trend is seen among cluster 2 patients. These distributions suggest that the current risk groups can be improved further with integrative clustering approaches. Another result that strongly supports this is the fact that our identified clusters give a much lower BIC and higher c-index compared to the existing HR / LR groups (1193 vs 1228).

We investigated the genes that are differentially expressed between the two clusters with *limma* method. The top 500 genes with the smallest adjusted *p*-values are listed in Additional file 2. Many of these genes have been previously found to be associated with neuroblastoma. The references are listed in the last column of the table. The top 50 genes are also plotted with heatmap (Additional file 3: Figure S1). We observe that these top 50 genes are expressed at higher levels in group 2 (High Risk).

**Fig. 5:**
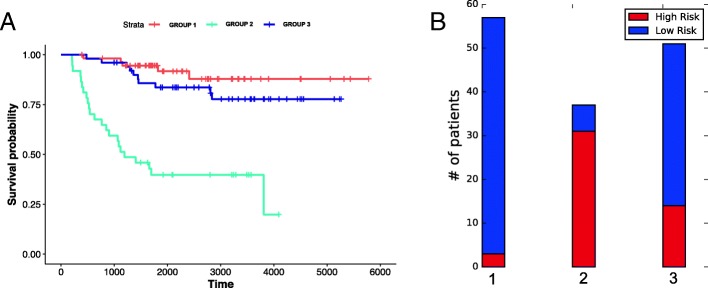
Analysis of the clusters obtained with both RNA-seq and microarray datasets **a** Kaplan-Meier analysis (log-rank test *p*-value: 1e-10) **b** Distribution of low risk and high risk patients in identified clusters

We repeated the same analysis with a subset of patients (i.e., 145 patients) that have aCGH data. Similar to what we observed in our experiments with all patients, combining the RNA-seq and the microarray datasets gives the best model (Table 5). Indeed, this is the only model where the discovered stratification is associated with a significant Wald-test *p*-value (i.e., 0.004). Interestingly, including aCGH data to this model resulted in no improvement. On the other hand, if we focus only on patients with no MYCN amplification (121 patients), including aCGH on top of gene expression datasets results in a slight improvement compared to using gene expression datasets only (BIC: 219 vs 220). For 145 patients, the MVKKM-learned weights for RNA-seq (MAV), RNA-seq (RPM) and microarray datasets were 0.30, 0.34 and 0.36 respectively. Figure 5a shows the survival plot of identified clusters. Here, we observe an intermediate risk group in addition to high risk and low risk groups. Indeed, the fraction of literature-defined LR patients increase as we go from our own high risk group to low risk group.

**Table 5:** Comparison of the clusters obtained with different data types from a subset of patients with aCGH data

Model		Coxphf			AFT	
Data	k	HR (confidence interval)	Wald-test *p*-value	BIC	rmse	c-index
aCGH	2	0.66 (0.32-1.35)	0.26	356	19.3	0.854
RNA-seq datasets + microarray	3	2.55 (1.32-5.10)	0.004	352	17.4	0.870
RNA-seq datasets + microarray (uniform)	2	0.80 (0.33-1.91)	0.61	357	19.1	0.857
RNA-seq datasets + microarray + aCGH	2	0.61 (0.29-1.26)	0.18	355	17.9	0.854

### Discussion

The availability of genome-wide datasets for cancer patients have increased rapidly in recent years. Methods that can effectively integrate these datasets can improve our understanding of cancer development and progression. To this end, we used supervised and unsupervised learning strategies to predict patient survival in neuroblastoma. Our supervised model can accurately predict overall survival and event-free survival profiles of neuroblastoma patients in independent cohorts. We evaluated models that are trained from RNA-seq or array-based gene expression data. Our experiments indicate that the differences in platforms of gene expression data between training and test cohorts may not be critical as RNA-seq-derived models are found to perform better than microarray-derived models for many of the prediction tasks where the test cohort contains array-based gene expression data. We observed that the prognosis of HR patients is harder to predict. One strategy to improve the prediction performance of HR patients is to focus on only HR patients in the training set.

To infer neuroblastoma subgroups by integrating different genomic characterizations of the patients, we utilized the MVKKM method. MVKKM has a number of advantages over other simpler clustering approaches. Namely, it uses an intermediate integration strategy that performs data integration and clustering steps concurrently. Besides, multiple data types can be combined with different weights enabling flexible and robust data integration. Finally a major advantage of MVKKM is the use of kernel k-means that enables nonlinear transformations to the feature space. We ran MVKKM on two sets of patients: (i) all patients; (ii) subset of patients with aCGH data and compared the different models that use distinct sets of data types with Cox proportional hazards regression results. Application of Firth’s correction to Cox regression was critical as we obtained completely different results without this correction (data not shown). Based on most of the evaluation criteria calculated from Cox regression and AFT models, combining all the available gene expression datasets resulted in the best model both for all the patients and for the subset of patients. For all patients, we discovered two clusters that have significantly distinct survival profiles. In parallel with the observed variability of prognosis within LR and HR patients, our identified clusters contain a mixture of LR and HR patients. For subset of patients with aCGH data, we discovered three clusters that correspond to low risk, intermediate risk and high risk groups. We observed that the proportion of literature-defined HR patients increases as the risk level of the group increases. Interestingly, addition of aCGH data on top of the gene expression datasets resulted in no improvement for this group of patients. This could be due to the high noise level of the aCGH data. The clusters that we identified in both patient groups indicates that the current stratification of patients to high risk and low risk groups can be improved via the integrated use of genomic datasets.

The performance of both supervised and unsupervised learning models can be further improved as genomic data becomes available for larger cohorts. Also, additional types of genomic data such as DNA methylation, protein expression or microRNA expression can provide opportunities to understand this complex disease from different angles and to pave the way toward improved choice of therapies. Indeed, a recent study discovered that the disruption of the let-7 microRNA family is an important mechanism in understanding cancer pathogenesis for neuroblastoma [29].

### Conclusion

Our study demonstrates that supervised learning models built from genomic datasets are suitable for clinical endpoint prediction in independent cohorts. Also, unsupervised integration of multiple genomic datasets with MVKKM reveals neuroblastoma subtypes with distinct survival profiles. Both supervised and unsupervised approaches can contribute to improved treatment stratification of neuroblastoma patients. Altogether, these results indicate that the use of multi-dimensional genomic datasets has the potential to improve current cancer risk models.

## Reviewers’ comments

### Reviewer’s report 1: Susmita Datta, University of Florida, Gainesville, USA

In this manuscript authors demonstrate that supervised learning models built from multiple genomic datasets are suitable for clinical endpoint prediction. Also, they use a form of Multi-view kernel k-means (MVKKM) algorithm to identify subtypes of Neuroblastoma which has distinct survival profiles. Both supervised and unsupervised approaches can contribute to improved treatment stratification of neuroblastoma patients. It is an interesting idea in include both supervised and unsupervised methods for the survival prediction. I have some major concerns:

**Reviewer comment:** Authors have used mean silhouette co-efficient to choose the number of clusters. Please note that there are many other indices to determine the quality of clusters such as Dunn-index and connectivity. One may be able to use them as well to determine the number of clusters. So a holistic way will be to use many such evaluation measures and rank them using methods such as Pihur et al., Bioinformatics, 2007 paper.

Author’s response: *We thank the reviewer for this suggestion. In addition to mean silhouette index, we now calculate the DUNN-index and connectivity for each value of k. Other measures mentioned in Pihur et al. were either meaningful only in the context of gene expression data; or they required substantial additional code for implementation. We used RankAggreg method proposed by Pihur et al. to combine the resulting rankings. The number of clusters chosen by the aggregation of these three methods remained the same in our experiments. As such, we did not update the results. However, we are glad that the aggregation method could improve the choice of number of clusters for future studies that focus on cancer subtyping.*

**Reviewer comment:** The wonder whether the assumptions of Cox proportional hazards model will work here or not. I do think Accelerated failure time model with penalty for high dimensional data would work better. So please comment why that wasn’t used.

Author’s response: *We think there is misunderstanding about how we run the Coxph model. We do not input high dimensional data (i.e., gene expression, aCGH etc) directly to the Coxph model. We first learn clusters from the kernel-transformed data and input these clustering assignments to the model together with age and tumor stage. However, what is critical is that we do account for the large proportion of censored samples with Firth correction. One advantage of using the Coxph model is that we can easily compare the hazard ratios that we obtained with those from literature. Nevertheless, we have also tried Accelerated failure time model where right censoring is handled by imputation as in Dr. Datta’s recent paper (Grimes et al. Biology Direct, 2018, 13:11). We now add adjusted root mean squared error (rmse) and Harrell’s c-index measures to Tables* 4*and* 5. *We do not report low-predicted survival (LPS) classification of patients as with the cutoffs* *t*=2*and* *t*=5, *we routinely got an error from survdiff function regarding the existence of only a single group. We think rmse and Harrell’s c-index are representative of model performance.*

In summary, even though the ranking of the models have changed in some cases, our main conclusions remained the same based on the results of the AFT model.

Also, during these experiments, we discovered a bug that leads to reading HR/LR labels incorrectly. As such, Figs. 4b, and 5b and the last row of Table 4 are updated accordingly.

**Reviewer comment:** How do you choose p related to sparsity in composite kernel?

Author’s response: *We thank the reviewer for this comment as we realized that we forget to include the choice of p in the* “Methods” *section. Our choice of p parameter is based on the following assumptions and prior results:*

- In this manuscript, we integrate datasets that are highly correlated (i.e., RNA-seq datasets normalized with two different procedures, or RNA-seq and microarray datasets). However, these datasets could still contain complementary information and have to potential to improve clustering when combined. As described in the manuscript, in MVKKM model, p=1 corresponds to the selection of only one view (i.e., dataset) and large values of p (e.g. *p*>=4) correspond to the case where views contribute uniformly to the composite kernel. We think that p values that lead to non-uniform weights would work best for our context.
- In Tzortzis et al. (ref 18), the authors have used MVKKM for several datasets and their results indicate that the choice of p within the range of {1.5-2.5} give the optimal results. Even though the datasets they used are of different context, we still think that the results are informative.
- We tried the values 1.5, 2 and 2.5 based on the discussion above. p = 1.5 was chosen according to mean silhouette coefficient. We now mention this in “Unsupervised learning” section.

**Reviewer comment:** What is the range of *p*?

Author’s response: *p has to be greater than or equal to 1. We state this in the second paragraph of* “Unsupervised learning” *section.*

### Reviewer’s report 2: Wenzhong Xiao, Massachusetts General Hospital, Cambridge, USA

In this manuscript, Baali etc. described their work comparing gene prediction models across multiple independent datasets of neuroblastoma and using genomic data integration modeling to study cell signaling mechanisms of high-risk neuroblastoma and to predict disease outcomes.

The paper touched upon a number of observations and issues that this reviewer thinks are important to the integration of multiple genomic data sets. However, more clarity is needed in the paper: while a reader can follow the methods and results described in the paper, some of the statements appear to be weak and confusing, and it is hard to draw conclusions.

**Reviewer comment:** A technical issue often came up in integration of multiple datasets is between data from arrays and data from RNA-seq. Tables 2 and 3 listed some of the results of comparing gene prediction models derived from the SEQC dataset on two independent test datasets for two endpoints and four settings. However, the results listed were sometimes models from arrays and sometimes from RNA-seq, making it hard for the readers to understand these results and the statements in the text comparing the results. For example, on Page 5 line 20–25, “Focusing only on HR patients in training improves the accuracy of predicting OS in HR patients since balanced accuracy increases from 0.71 to 0.79. However, we do not see the same effect for predicting EFS, as the accuracy decreases if we train only with HR patients.” And on Page 5 line 60–62, “The prediction accuracy of the HR patients increases when training is performed only with HR patients (comparison of rows 2 and 3 in the table).” Clarification is needed since models compared here were derived from data of different platforms.

Author’s response: *We thank the reviewer for raising this important point. We checked whether our observations still hold when we compared the models that use data from the same platform. The balanced accuracy and AUROCs of all the models are now listed in Additional file* 4.

We changed the result section accordingly. In particular, in section “Predicting outcome in Versteeg cohort” section we added the following text:

*Here we should note that some of the models listed in Table* 2*use gene expression data derived from different platforms (e.g. RNA-seq or microarray). As such, we checked whether these observations still hold if we restrict the comparison to models that use gene expression data from the same platform* (*Additional file* 4). *The optimal models identified for OS and EFS prediction in*$ All\rightarrow All $*and*$HR\rightarrow HR $*contexts use gene expression data from the same platform whereas this is not the case for*$ MYCN\_NA \rightarrow MYCN\_NA $*context. For*$MYCN\_NA \rightarrow MYCN\_NA $*context, if we compare models that are derived from the same platform only, we still observe that EFS prediction has better performance than OS prediction.*

and

*To confirm that the differences in accuracy between* $ All\rightarrow All $ *and* $ HR\rightarrow HR $ *contexts are indeed due to the different training datasets used, we repeated the comparison with models that use gene expression data from the same platform. When RNA-seq based gene expression data is used, models that are learned from only HR patients perform better than models that are learned from all patients in predicting OS of HR patients. However, we do not observe the same improvement for microarray based models.*

Similarly, in section “Predicting outcome in TARGET cohort” section, we added the following sentence:

*When we restrict the comparison to models that use the same type of gene expression data, models derived from RNA-seq data predict both OS and EFS of HR patients more accurately when the training was performed with HR patients only* (*Additional file* 4). *Unlike our results on Versteeg cohort, predicting EFS is more accurate than predicting OS in terms of balanced accuracy. The optimal models identified for OS and EFS prediction in*$ All\rightarrow All$*context use gene expression data from the same platform whereas this is not the case for*$ HR \rightarrow HR $*and*$ MYCN\_NA \rightarrow MYCN\_NA $*contexts. For these two contexts, balanced accuracy of predicting EFS is higher than that of predicting OS even when we compare models that are derived from the same platform* (*Additional file* 4).

Note that the comment below is about the training set performance of the same model with and without the balanced option – for a particular context and prediction type (OS or EFS). As such it does not require us to compare models of the same gene expression platform.

*Turning on the balanced option for class weights gives a better model in terms of training set performance except for EFS prediction in* *All*→*Allmodel. This could be due to the fact that the EFS label is less unbalanced than the OS label in the SEQC cohort.*

**Reviewer comment:** On Page 6 line 7–11, the author stated that “we see that the microarray-based models are preferred over RNA-seq. This could be due to the fact that the microarray platform is used to measure gene expression in the TARGET cohort”; is this true for the Versteeg cohort as well, since it used microarrays as well? Would the models from arrays show the best performance in general? It would help if the authors can either discuss the test results of the models derived from each platform, or show these results as supplemental information.

Author’s response: *We thank the reviewer for this comment. Indeed, we have realized that the same observation does not hold for the Versteeg cohort where gene expression data is also array-based. As such, we have now removed those sentences from the* “Results and discussion” *section and instead included the following sentences to Discussion.*

*Our experiments indicate that the differences in platforms of gene expression data between training and test cohorts may not be critical as RNA-seq-derived models are found to perform better than microarray-derived models for many of the prediction tasks where the test cohort contains array-based gene expression data.*

**Reviewer comment:** As shown in Table 3, the performance of the predictive models was dramatically lower in the TARGET cohort. The authors mentioned that the TARGET cohort had a high fraction of HR patients, suggesting that the prediction of outcomes of HR patients is much more difficult. This should be emphasized in the text and begs the question on the performance of predicting the outcomes of these HR patients when applying MVKKM on the data.

Author’s response: *In addition to the* “Results and discussion” *section, we also mention the difficulty of predicting the outcomes of HR patients in the first paragraph of the* “Discussion” *section.*

We could not understand the second part of this comment as we have not applied MVKKM on TARGET cohort. Since MVKKM is an unsupervised approach that outputs clustering information of patients, it is unclear to us how it could be used to predict patient outcomes directly.

**Reviewer comment:** Besides, on Page 2 line 55–58, “Chen Suo and her colleagues have identified a potential mislabeling of 32 neuroblastoma patients in aCGH data (personal communication). As such, we updated the aCGH data accordingly.” The authors then stated that the aCGH data did not improve the results of prediction. Can the authors reference the information or include details of the corrections they made so readers can potential reproduce the results?

Author’s response: *We now included a Additional file* 2 (*Additional file* 2) *that lists the ids of 32 patients and explained the correction in more detail in text as follows:*

*Chen Suo and her colleagues have identified a potential mislabeling problem between normal and tumor samples when they compared the aCGH data against the MYCN status derived from FISH experiments (personal communication). Based on this comparison, the sign of the intensity values for 32 patients were reversed. Ids of these patients are listed in Additional file* 2.

**Reviewer comment:** Figure 3 does not seem to be referenced in the text. 2. In the PDF file of the manuscript, there are a number of warnings (page 2 - 21) Author’s response: *Fig.* 3*is now referenced in the beginning of the section.*

### Reviewer’s report 3: Ziv Shkedy, Hasselt University, Belgium

**Reviewer comment:** The paper describe supervise and unsupervised methods for the development of multi- source signature for High risk /Low risk survival patients. It is an applied paper which presents an analysis for different datasets. The paper is interesting but poorly written and a strong language editing is needed in order to transform the current text to a level of a scientific publication (which is not, to my opinion, the level of the current version of the manuscript). I listed below few examples (there are much more).

Author’s response: *We thank the reviewer for detailed suggestions on language usage. We made the necessary changes for all the items listed below. Detailed replies are available below. Additionally, we revised several other parts of the manuscript to improve clarity. We hope that our revised manuscript reads better.*

**Reviewer comment:** Page 2, line 28: “Using a linear SVM” should be “Using a linear Support vector machine (SVM, ref)”. See for example, line 37.

Author’s response: *We have fixed this now and inserted the reference for SVMs.*

**Reviewer comment:** Page 2, line 55 “.....CGH datasets for the SEQC cohort”. You should use the same font for SEQC, see for example page 4 lines 38, 48 etc. Author’s response: *We have fixed the font and used the full name for the SEQC acronym.*

**Reviewer comment:** Page 3, line 25: “the SVC function available”, SVC mentioned for the first time, use the full name and give reference.

Author’s response: *We have spelled out the full name for SVC and inserted its reference.*

**Reviewer comment:** Page 3, line 35: "the C parameter" it is not clear what the C parameter is.

Author’s response: *We have now inserted a description for the C parameter.*

**Reviewer comment:** Page 3, line 47 “Multi-view kernel k-means (MVKKM)”, you do not need to use the ?full name since it was already mentioned in the abstract and in page 2, line 47.

Author’s response: *This has been corrected.*

**Reviewer comment:** Page 3, line 60-page 4 line 5: All parameters of equation (1) should be in ONE sentence with “,” between the parameters.

Author’s response: *The parameters are now explained in a single sentence.*

**Reviewer comment:** Page 4, line 23: add space before “where”.

Author’s response: *This has been corrected.*

**Reviewer comment:** Page 4, lie 52: the title of the subsection should not be numbered 0.0.1.

Author’s response: *We have removed the numbering before the subsection title.*

**Reviewer comment:** Page 4 line 57-page 5 line 8: this text should be a part of the caption of Table 2 and not a part of main text.

Author’s response: *We have moved the model definitions to the caption of Table* 2.

**Reviewer comment:** Page 5, line 43: “We also plot the survival profiles of these different approaches in Fig. 2” should be: “Fig. 2 shows the survival profiles the different approaches indicates that the model that is trained entirely on another cohort (i.e., SEQC) performs remarkably well on predicting OS in Versteeg cohort.”

Author’s response: *This is now fixed. Thank you.*

**Reviewer comment:** Page 6, line 43: “We also performed the experiments.....” should be “The experiments was performed in the opposite...”. In general try to reduce the number of times that you write “We also plot.....”, “we also compared...”, “ we also investigated...”, etc.

Author’s response: *We have edited the entire manuscript based on this comment and reduced the number of sentences that start with we.*

**Reviewer comment:** Page 7, line 29: “silhouette score” is mentioned for the first time, it is not clear what is it, add a ref.

Author’s response: *Silhouette score is already defined in page 4, we have now included a reference for it.*

**Reviewer comment:**Page 7, line 48: “The log-rank test *p*-value of this analysis confirms that the patients in the two clusters show distinct prognosis (i.e., 3.9e-20).” should be “The log-rank test *p*-value is equals to XXXX confirms that the patients in the two clusters show distinct prognosis.”

Author’s response: *This has been corrected.*

**Reviewer comment:**Page 7, line 60 “adjusted *p*-values” is mentioned for the first time. Based on which methods the p values are calculated?

Author’s response: *We have defined adjusted p-values in Methods and included the multiple-testing correction method.*

**Reviewer comment:** Page 7, line 61, give a reference to the limma method.

Author’s response: *The reference for the limma method is already provided in the* “Methods” *section.*

**Reviewer comment:** Page 9, line 36: Abbreviation should be excluded. Give the Abbreviation for each method in the text in the first time that it is mentioned. For example, see page 2, line 27 for MVKKM.

Author’s response: *Abbreviation section is inserted due to the journal instructions.*

**Reviewer comment:** Page 13, Tables 1, 2, 3: delete form the captions “This table shows details about the..”, This table summarizes the..”. “ This table summarizes the” and instead give titles. For example: in Table 1, use you can “Patients and the data types for the cohorts: SEQC, Versteeg, TARGET. Note that...”

Author’s response: *We have now rephrased the table captions to include titles.*

Some of the other changes we made are listed below:

- We have converted numeral representations to words to express the numbers below 10.
- The captions for Tables 4 and 5 are revised to emphasize the difference in datasets.
- We have converted a number of sentences in the “Methods” section to passive voice to limit the number of sentences that start with “we”.
- The full names for SVM and MVKKM are removed in the “Results and discussion” section as it is already introduced in Introduction.
- All occurrences of the word Versteeg and SEQC are now italicized.
- The abbreviation HR is introduced after the first use of High Risk.

## Additional files

Additional file 1 List of patients for which aCGH data is corrected. This text file contains the list of 32 patients for which aCGH data intensities are reversed. (TXT 191 b)

Additional file 2 Differential Expression Results. This spreadsheet contains the results of *limma* analysis on RNA-seq-MAV data. The data for the top 500 genes with smallest adjusted *p*-values are included. The columns indicate gene ID, log fold change, average expression of the gene, t-statistic, *p*-value, adjusted *p*-value, B-statistic and a column that indicates whether this gene has been found to be associated with neuroblastoma in literature. (XLS 110 kb)

Additional file 3 **Figure S1.** Heatmap of differentially expressed genes. The heatmap plots the expression (RNA-seq-MAV) of the top 50 genes with smallest adjusted *p*-values for the two clusters. (PDF 669 kb)

Additional file 4 Performance metrics of models for predicting OS and EFS in *Versteeg* and *Target* cohorts. This spreadsheet lists the performance metrics of models trained from SEQC cohort. Models that use different types of gene expression data (i.e., microarray, RNA-seq (MAV), RNA-seq (RPM)) are listed individually. (XLSX 40 kb)

## Abbreviations

aCGH: Array comparative genomic hybridization
AFT: Accelerated failure time models
AUROC: Area Under the receiver operating characteristic curve
BIC: Bayesian information criterion
CAMDA: Critical assessment of massive data analysis
Coxphf: Cox proportional hazards regression model with Firth’s correction
EFS: Event-free survival
HR: High Risk
LR: Low risk
MVKKM: Multi-view kernel k-means
OS: Overall survival
RBF: Radial basis function
rmse: Root mean squared error
SEQC: Sequencing quality control consortium
SVM: support vector machine
