# Supplementary figures and images for: Predicting clinical outcomes in neuroblastoma with genomic data integration
Source: Biol Direct. 2018 Sep 27;13:20. doi: 10.1186/s13062-018-0223-8 (PMC6889397; doi:10.1186/s13062-018-0223-8)

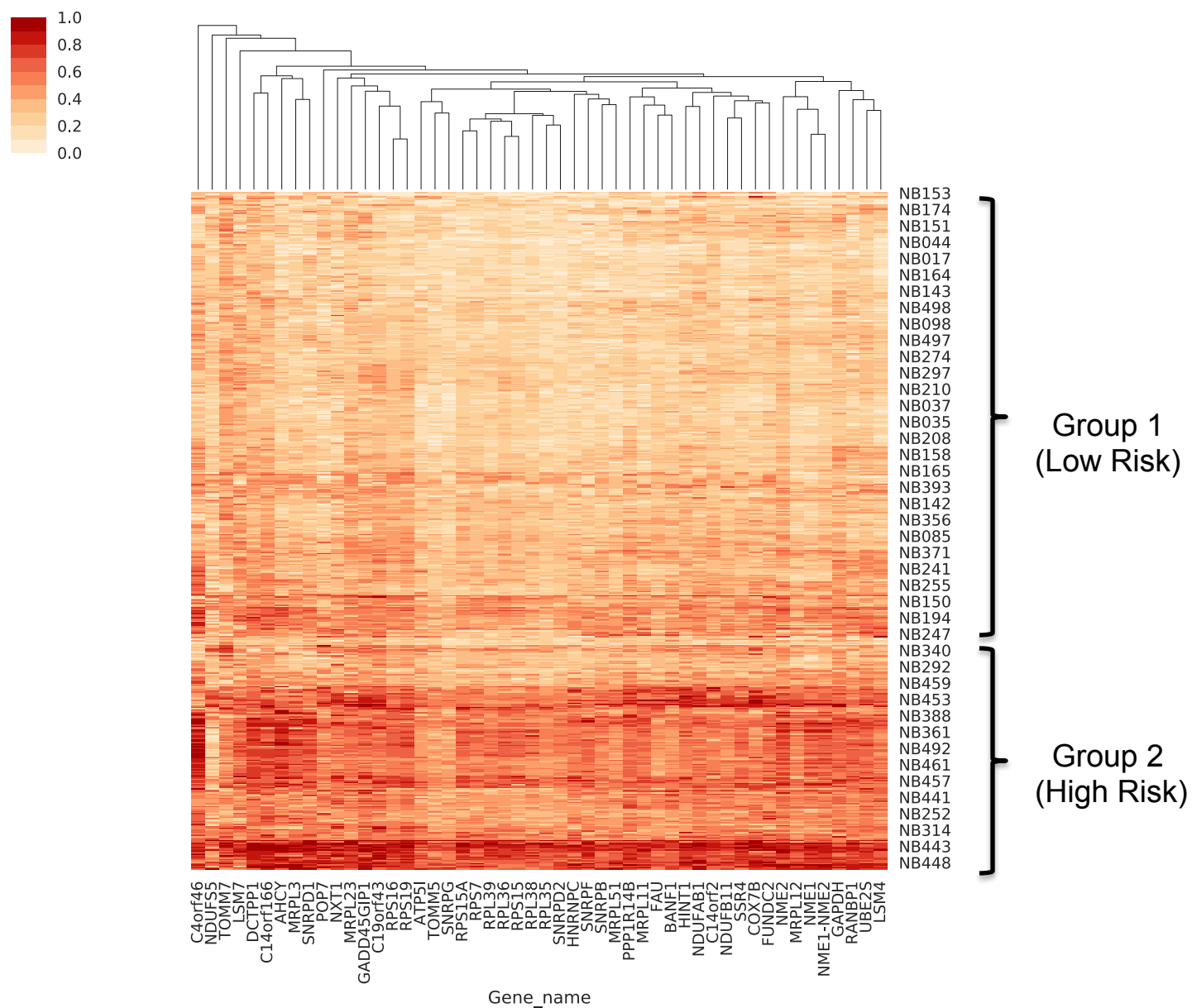

Supplement: Supplementary file 1 — List of patients for which aCGH data is corrected. This text file contains the list of 32 patients for which aCGH data intensities are reversed. (TXT 191 b) [file 13062_2018_223_MOESM1_ESM.pdf]
